# Supplementary material for: Are carbon emissions trading and green financial instruments synergistic? -Comprehensive quantitative research based on content analysis
Source: PLoS One. 2024 Mar 7;19(3):e0298601. doi: 10.1371/journal.pone.0298601 (PMC10919601; doi:10.1371/journal.pone.0298601)
Supplement: S1 Data — (ZIP) [file pone.0298601.s001.zip › Data/地级市Z_F工作报告.zip!/╣π╢1⁄2/╖≡╔╜╩╨/└┤╫╘╣1⁄2╓┌║┼í╛┬φ┐╦╩2╛▌═°í┐_2006.docx]

各位代表：

　　 我代表佛山市人民政府向大会作政府工作报告，请予审议，并请政协委员和其他列席人员提出意见。

“十五”时期及2005年工作回顾

“十五”时期的五年，是我市改革开放和现代化建设取得重大成就的五年。五年来，市政府坚持以邓小平理论和“三个代表”重要思想为指导，树立和落实科学发展观，贯彻落实党的十六大、十六届三中、四中、五中全会精神，围绕市第九次党代会确定的目标，以发展为主题，以行政区划调整为契机，全面推进产业强市、文化名城、现代化大城市与富裕和谐佛山建设，认真贯彻中央关于加强和改善宏观调控的决策，成功抗击非典型性肺炎、高致病性禽流感疫情和超百年一遇特大洪水袭击，经济保持了快速健康协调发展，社会各项事业取得新成绩，城市综合竞争力得到提升，市十一届人大四次会议通过的“十五”计划各项指标顺利完成。

——经济持续快速稳定增长，经济实力跃上新台阶。“十五”时期第一年，生产总值突破1000亿元大关，预计2005年达2379.8亿元，增长19.2%，“十五”时期年均增长14.9%（以下简称年均增长）；人均生产总值41031元（按常住人口计算）。经济运行质量效益持续改善，主要经济指标全面和超额完成。预计2005年农业总产值163.94亿元，增长4.4%；工业总产值5464.05亿元，增长24.9%；地方财政一般预算收入130.85亿元，增长18.28%；税收总额369.64亿元，增长15.49%；全社会固定资产投资753.26亿元，增长33.2%，“十五”时期累计完成固定资产投资达2290.43亿元；社会消费品零售总额634.47亿元，增长17%，年均增长13.5%。

——行政区划调整和体制机制创新，增强加快发展的新活力和动力。2002年12月我市进行了行政区划调整，实行新的行政管理体制,原有的“活力”和调整赋予的新“合力”得到充分发挥。城市资源有效整合。整合路桥收费站、城市公交、邮政、电信、旅游、教育和传媒资源，基本实现“同城生活，同城便利”。行政管理体制改革持续推进。实行简政放权，先后向各区下放322项行政管理事项。推进行政审批制度改革，市和各区设立了行政服务中心，提供“一站式”服务，实行“并联式”审批。顺利完成市、区两级政府机构改革，进行了镇（街）、村调整，全市镇（街）、村撤并率分别达到42.85%和20.6%。农村改革实现新突破。出台并逐步实施“农村工作五十条”及首批九个配套文件，农村社会保障体系、农村居民培训和就业制度初步建立。村级财务引入中介组织管理改革试点和深化农村股份合作制改革等工作进展顺利，农民专业合作经济组织进一步完善。2004年起免征农业税，减轻了农民负担。公有企业改革稳步推进。建立国有资产管理机构，加大产权多元化改革力度，企业改革重组进展顺利，劣势企业退出市场工作取得较大进展。金融体制改革有力推进，市商业银行被兴业银行成功并购，农村信用社改革顺利展开，利用资本市场步伐加快。投融资体制改革不断加强。出台并实施《佛山市推进城市可经营项目投资多元化市场化的指导意见》，一批城市可经营项目实行市场化经营。举办了城市可经营项目投资推介洽谈会，共签约项目73项，投资经营总额229.78亿元。研究制定城市可经营项目监管办法，确保公共产品安全和有效供给。社会管理体制改革不断深入。各类社会中介组织得到培育和发展，一批产业性行业协会、商会得到整合和规范。建立国库集中支付制度，严格执行“收支两条线”规定。各区公务用车制度改革顺利完成。

——产业强市、文化名城和现代化大城市建设全面推进，综合实力有了新提升。产业强市建设取得新成效。全力推进园区整合和园区基础设施建设，加快产业结构调整步伐，促进产业集群发展。工业结构不断优化，呈现出适度重型化、高级化趋势，规模以上轻重工业比例由2000年的1∶ 0.7调整为2005年的1∶ 0.97。农业产业化现代化水平不断提高，农产品质量安全管理工作取得新进展，建立农业标准化示范区7个，无公害生产基地88个。第三产业发展水平不断提高，预计“十五”期末第三产业增加值858.58亿元，增长10.9%，年均增长11.2%；全市物流业增加值400.95亿元，占第三产业增加值的46.7%。文化名城建设取得新进步。成功承办亚洲文化部长论坛暨第七届亚洲艺术节、粤剧琼花艺术节，举办武术文化节、中国陶艺文化节等系列活动，城市内涵和品位得到提升。文化旅游设施建设加强，建立了以文化设施为载体的四级文化网络。民间文化艺术和文物得到有效保护和利用，文化、旅游产业进一步发展。全力推进科教兴市、人才强市战略，加强整体性人才资源开发，为建设产业强市、文化名城和现代化大城市提供智力支持和人才保障。我市被评为全国科技进步先进市和被确定为全国专利工作试点城市。现代化大城市建设取得新进展。坚持以规划引领城市建设和发展，编制了城市发展概念、市域城镇体系、干线公路网、中心组团近期建设和可持续发展生态环境等一批总体规划、专项规划和分区规划，规划体系逐步建立，规划管理技术标准逐步统一。落实省城镇化试点工作要求，建立城市规划管理新体制。全面启动构建现代化大城市框架和雏形的“十大工程”建设。一批以中心组团新城区为重点的城市基础设施和以干线公路为骨架的道路网络正在建设。以创建国家环保模范城市和全面创建国家卫生城市为契机，大气污染治理、内河涌和珠江综合整治工作取得初步成效，城市面貌得到改善，城市绿化水平明显提高，生态示范区建设进一步加强。创建国家卫生镇10个、省卫生镇20个、省卫生村313个、省级生态示范区（镇、园、村）35个。

——社会各项事业全面进步，人民生活水平有了新提高。预计“十五”期末城市居民人均可支配收入17400元，增长8.4%；农村居民人均纯收入7569元，增长6.8%；城乡居民储蓄存款余额2319.52亿元。“五网络一中心”的社会安全网络初步建立，社会治安管理基础进一步夯实，开展严打整治斗争，扫除“黄赌毒”等社会丑恶现象，加强对“法轮功”等邪教活动的防范打击，国家安全工作进一步加强，社会大局稳定。做好全国非公有制中小企业安全生产监管试点和推广工作，逐步建立安全生产长效机制，交通、消防和森林防火安全态势平稳。重视做好人民调解和群众来信来访工作，加强政府机关与传媒互动机制建设，切实关注民生。大力整顿和规范市场经济秩序，社会信用体系建设步伐加快。全面推进依法治市建设，完成“四五”普法工作；贯彻实施《行政许可法》，落实行政执法责任制，行政综合执法工作顺利推进。出台《佛山市国家公务员服务行为规范暂行规定》，严格考核制度，政府依法行政水平和公务员素质不断提升。全面贯彻《公民道德建设实施纲要》，组织开展“爱国、守法、诚信、知礼”现代公民教育活动，市民的思想道德素质和文明程度进一步提高。群众性精神文明建设活动深入开展，我市创建为“广东省文明城市”。国防建设和民兵预备役、拥军优属工作得到进一步巩固和提高，我市第五次荣获“全国双拥模范城”称号。

与此同时，人口与计划生育、残疾人事业、妇女儿童事业、粮食储备、民族宗教、外事侨务、对台事务、人防、气象、保密、档案、物价、统计、老龄和对口扶贫等各项工作都取得新成绩。

2005年，我们按照科学发展观的要求和市委九届七次全会以及市十二届人大三次会议确定的经济社会发展目标，“抓实机遇、紧盯目标、乘势而为、上新水平”，抓落实、重提高、促发展，经济和社会各项事业取得较大成效，市十二届人大三次会议确定的各项预期目标已经实现，为“十五”计划完成画上了圆满句号，为“十一五”规划开好局奠定了良好基础。

一、以科学发展观为指导，推动经济全面协调可持续发展

大力推进集约园区建设。初步建立引导园区向集约化发展的考核评估体系，推进园区基础设施建设和园区认证工作，园区环境不断改善。高明沧江工业园和三水中心工业区获得质量管理、环境管理和职业健康与安全体系三项认证。预计工业园区和产业基地实现工业总产值2300亿元，增长32.8%。

加快产业结构调整优化升级。以专业镇为载体，通过推广西樵纺织产业升级示范区、禅城区不锈钢和陶瓷产业资源整合经验，推动陶瓷、家电、纺织和有色金属等传统产业改造提升，成为省级产业集群升级示范区；汽车配件等新兴产业加快发展。

积极推进企业技术创新和名牌战略实施。顺德、南海、禅城在重点行业中建立了创新平台，全市的龙头企业和名牌产品不断涌现，新增中国驰名商标2个，中国名牌产品10个。

进一步提高外经贸工作的质量和水平。创新外贸发展方式，出口结构进一步优化。预计进出口总值253.42亿美元，增长16.9%，其中，出口总值168.22亿美元，增长21.6%；合同利用外资15.2亿美元，实际利用外资8.9亿美元，分别增长31%和28%。完善“三大机制一支队伍”的招商引资机制，招商引资质量不断提高。新增世界500强投资项目12个，增资6个，目前已有33家世界500强企业在佛山投资了56个项目。企业增资取得新进展，增资金额7.54亿美元，增长54.65%。

大力发展以物流业为重点的现代服务业。成功举办第二届佛山（国际）物流合作洽谈会，签约项目84项，签约金额184.07亿元。澜石国际金属物流交易中心、百佳珠三角城市配送中心、华南（国际）物流钢铁交易中心等一批投资规模大、辐射能力强的项目相继投入运营，一批第三方物流企业发展壮大。积极推动连锁经营等新型业态发展，沃尔玛、吉之岛等国内外品牌连锁企业在我市设点。新协力集团有限公司、西樵轻纺城有限公司荣获首批“中国商业名牌”企业。

优化民营经济发展环境。认真贯彻国务院《关于鼓励支持和引导个体私营等非公有制经济发展的若干意见》，进一步抓好环境的优化，加快信用担保等服务体系建设。大力推广“美的”发展经验，引导和鼓励民营企业与外资结合。加大对民营企业技术改造和科技创新的扶持力度，鼓励和引导民营企业创立品牌，提升核心竞争力。预计全市规模以上民营企业实现工业产值2466亿元，增长31%，占全市规模以上工业总产值的52.5%。

做好节约型社会试点工作。贯彻落实国务院关于做好建设节约型社会工作的要求，按照省的部署，积极开展建设节约型城市试点的工作。

二、以规划为龙头，推进重点工程和组团城市建设

抓好规划的编制、衔接和实施。编制完成《佛山市城市总体规划》、《佛山历史文化名城保护规划》、《佛山市城市绿地系统规划》、《“一环”快速干线沿线地区规划与城市设计》和《中心组团新城区修建性详细规划》等，按照建设“2+5”组团城市的发展战略，全力推进各项规划的实施，加强规划监管，确保各区城市规划、各专项规划与城市总体规划的衔接与协调。

加速推进以“一环”为重点的交通基础设施建设。21项主干路网工程有8项已建成通车，“一环”完成路基、桥涵总工程量的80%。以325、105国道改造为重点的16项工程相继完工，高速公路和航道建设工程进展顺利。

加快亚艺节、省运会和中心组团新城区等重点工程建设。顺德演艺中心、琼花大剧院和亚洲艺术公园等工程如期竣工，岭南明珠体育馆、世纪莲体育中心、游泳馆等工程按计划推进，中心组团新城区启动区基础设施建设全面展开。全力推进创模工程建设。城乡水环境综合整治、电厂脱硫、陶瓷和纺织等重点行业污染治理、市容市貌整治等初见成效，全市建成污水处理厂14座，污水处理能力达64.5万吨/日。推行固体废物规范化管理，建成高明无害化生活垃圾填埋场，生活垃圾无害化处理率达60%。抓好电力、电网建设与改造，建成一批电网工程。抓好城乡水利三年防灾减灾工程建设，努力建设比较完备的防洪减灾保障体系。

认真抓好交通资源整合和信息化建设。深化年（次）票制度改革。出台扶持措施，促进公交企业做强做大。优化各组团中心城区公交线路，推进广佛公交无缝对接。以应用为龙头，认真抓好信息化建设，信息化水平不断提高。预计信息化综合指数达80.5%。

三、以承办亚艺节和争创教育强市为契机，促进文化名城建设跃上新台阶

成功承办亚洲文化部长论坛暨第七届亚洲艺术节。使之成为“艺术的盛会、人民的节日”，达到“安全、热烈、精彩、圆满”效果，提升了佛山的城市形象和文化内涵。

成功创建“广东省教育强市”。完善九年义务教育“以区为主”的教育管理体制。不断巩固基础教育，大力发展职业教育，加快发展高等教育，扎实推进社区教育，积极扶持民办教育发展，以信息化带动教育现代化，教育综合实力进一步增强。

全力推进科技创新工作。新增省级高新技术企业89家，国家火炬计划重点高新技术企业（集团）5家，省级工程中心5个，省级专业镇7个，预计高新技术企业产品产值1270亿元，占工业总产值的23.2%。产学研合作进一步推进，高校和科研院所在我市新增2个办事处，1个院士工作室。知识产权工作成效显著，共申请专利14500多件，专利授权8000件，分别增长34.4%和29.2%。

积极实施人才强市战略。进一步优化人才环境，创新人才政策，加大中高级人才引进与培养力度，积极为用人单位引进各类人才。2005年全市共引进各类在职人才2127人，其中高级职称人员211名，海外留学人员30名。目前，全市人才总数达55万人，增长5%。

加快卫生、体育和人口与计生事业发展。公共卫生体系建设得到加强，形成覆盖全市的公共卫生三级网络；农村和社区卫生、妇幼保健、科技兴医等工作取得新进展；推行市场准入管理，初步建立起药品、食品、农产品卫生安全监管机制。成功举办第六届市运会，竞技体育水平跃上新台阶。第十二届省运会各项筹备工作顺利推进，群众性体育活动蓬勃开展。落实人口与计生目标管理责任，全市户籍人口出生率为9.97‰，自然增长率为4.88‰。

四、以落实“十项民心工程”为重点，推动和谐佛山建设

抓好就业和再就业工作。城镇失业人员再就业4.08万人，就业率达59.8%；期末城镇登记失业率2.13%。城乡培训就业一体化进程加快，共培训农村富余劳动力3.32万人，推荐就业1.38万人，就业率为41.5%。

推进社会保障体系建设。年末基本养老、失业、基本医疗、工伤保险参保人数分别达到134万人、100万人、114万人、137万人。农村基本医疗保险制度全面推行，农村参保人数204万人，参保率97%；全征地农村居民养老保险补贴覆盖面进一步扩大，建立扶贫助学机制，妥善解决低保家庭子女读书难问题，城乡居民最低生活保障实现“应保尽保”。

抓好社会稳定工作。进一步强化安全生产责任制，狠抓安全生产、消防安全专项检查和重大隐患的整改工作，工矿商贸企业职工伤亡事故宗数比去年同期下降26%，死亡人数与去年同期持平。全国科技强警示范市建设取得阶段性成果并通过验收考评。积极构建社会面立体防控网络，深入开展平安和谐社区创建活动，确保人民群众安居乐业。健全企业工资支付监控制度，大力清理拖欠工程款和农民工工资，完成政府拖欠工程款清理工作。进一步完善法律援助制度，为外来工提供法律援助。

五、以提高理政能力为目标，进一步加强政府自身建设

行政管理体制改革继续深化。推进新一轮行政审批制度改革，清理各种审批事项1400项，其中取消157项。统一规范和公示全市审批事项的办事程序，建立健全行政审批管理监督制度。电子政务建设不断加快，政务公开工作稳步推进。“一站式”服务进一步完善，初步建立“一市五区”行政服务互通机制。完成公安、国土、文化、广播电视、新闻出版等部门的机构改革任务，加强安全生产监督管理体系建设。大力扶持中介组织和行业协会发展。

应对突发事件能力明显增强。群体性和突发性事件处置能力加强，社会援助联动机制和城市公共安全应急机制加快建立，各项应急预案进一步完善和落实，公共安全保障水平不断提高。

民主法制和廉政建设不断加强。坚持向人大及其常委会报告工作，向政协通报情况，加强与各民主党派、工商联及工青妇等人民团体的联系。全年共办理人大代表议案56件、建议50件和政协提案191件。贯彻落实《全面推进依法行政实施纲要》，完善和规范行政执法工作。加强政风建设，充分发挥监察、审计等监督机关的职能作用，强化行政执法监察和效能监察。落实“为民、务实、清廉”要求和党风廉政建设责任制，规范领导干部廉洁从政行为，从严执法执纪，严肃查处违纪违法案件。

此外，抓好《佛山市国民经济和社会发展第十一个五年规划纲要》编制工作。

各位代表，“十五”时期的五年是我市综合实力、城市形象进一步提升，改革开放和现代化建设取得辉煌成就的五年。这些成绩的取得，是在党中央、国务院和省委、省政府以及市委的正确领导下，全市人民与时俱进、团结奋斗、开拓创新、扎实苦干的结果，是市人大及其常委会和市政协监督支持的结果，是中央、省驻佛山单位和社会各界关心支持的结果。在此，我代表佛山市人民政府，向辛勤工作在各条战线的广大干部群众，向给予政府工作大力支持的人大代表和政协委员，向中央、省驻佛山单位，向驻佛山的人民解放军、武警部队官兵和各民主党派、工商联、人民团体、社会各界人士以及所有参与、关心和支持佛山建设的港澳台同胞、海外侨胞和国际友人表示衷心的感谢！

通过“十五”时期的成功实践，我们不仅推进了经济社会各项事业向前发展，而且在丰富的实践中升华了思想认识，增强了贯彻党中央、国务院和省委、省政府决策部署的自觉性，提高了用好重要战略机遇期、更好推动发展的认识和能力。我们的主要体会是：必须坚持以邓小平理论和“三个代表”重要思想为指导，以科学发展观统领经济社会发展全局，解放思想，实事求是，与时俱进，开拓创新，结合佛山的实际创造性地开展工作。在具体工作中，做到“八个坚持”：一是坚持“发展才是硬道理”，牢记使命责任，抢抓机遇，加快发展。二是坚持围绕大局，服务中心，凝聚和调动各级各方面的积极性和创造性，推动经济社会全面协调可持续发展。三是坚持规划引领，不断提升经济社会发展和城市建设水平。四是坚持与时俱进，不断创新和转变经济增长方式。五是坚持以改革为动力，优化发展环境，增强发展活力和动力。六是坚持转变政府治政理念，为基层加快发展营造优质服务环境。七是坚持以人为本，推进和谐佛山建设。八是坚持教育制度监督并举，抓好队伍廉政建设。

在肯定成绩的同时，我们也清醒地看到，我市经济和社会发展仍然存在不少困难和问题，主要表现在：经济结构依然不够合理，自主创新能力有待加强，产业发展集约化水平不高；经济社会健康发展仍面临不少体制机制性障碍；经济社会发展与资源环境的矛盾日益突出，可持续发展能力有待提高；“三农”问题仍有不少深层次矛盾需要解决；城市建设和管理滞后于经济社会发展，现代化大城市意识有待增强，人文环境有待进一步改善；政府治政理念和职能转变还有不适应的地方；和谐佛山建设还有不少隐忧等等。对于这些问题，我们一定高度重视，在“十一五”时期采取有力措施认真加以解决。

“十一五”时期奋斗目及2006年工作安排

“十一五”时期是我市进一步落实科学发展观，全面推进产业强市、文化名城、现代化大城市与富裕和谐佛山建设的关键时期。

“十一五”时期，我市经济社会发展面临难得机遇。从国际方面看，随着我国加入WTO后过渡期即将结束，市场开放度进一步扩大。新一轮国际资本流动和产业转移趋势，有利于我市引进先进技术和高端产业，为我市提高利用外资质量和水平，促进产业结构优化升级带来难得机遇。从国内方面看，本世纪头二十年是我国发展的重要战略机遇期。我国经济增长方式加快转变，社会主义市场经济体系不断完善，区域发展加速融合，为我市经济保持持续快速健康发展提供有利机遇。从我市来看，目前已形成较强的经济实力，具备加快发展、率先发展、协调发展的良好基础和内在动力，但也面临“不进则出局、小进即落伍”的严峻挑战以及经济社会发展的一些深层次矛盾和问题。因此，我们要站在新的历史起点上，认清形势，紧盯目标，抓实机遇，乘势而为，推动经济社会乘势发展、集聚发展、创新发展，把改革开放和现代化建设提高到一个新高度。

根据党的十六大、十六届五中全会和省委九届七次、八次全会精神，以及市委九届八次全会关于“十一五”规划《建议》的要求，市政府组织编制了《佛山市国民经济和社会发展第十一个五年规划纲要》，提出了“十一五”时期我市经济社会发展的指导思想和奋斗目标。

指导思想是：以邓小平理论和“三个代表”重要思想为指导，以科学发展观统领经济社会发展全局，以加快发展、和谐发展为主线，以建设产业强市、文化名城、现代化大城市与富裕和谐佛山为总目标，立足以人为本，转变经济增长方式，优化产业结构，提高发展质量，着力自主创新，完善体制机制，发挥整体优势，促进社会和谐，全面增强城市综合实力和竞争力，把我市经济建设、政治建设、文化建设和社会建设提高到一个新水平。

奋斗目标是：经济综合实力进一步增强，全市生产总值年均增长12%，到2010年实现全市人均生产总值比2000年翻一番以上；单位生产总值能源消耗比“十五”期末降低20%左右；自主创新能力明显增强，初步建成产业布局合理、先进发达的制造业基地；“2+5”组团城市基础设施基本成型，生态环境改善明显，文化名城特色鲜明；体制机制不断完善，各项事业繁荣发展，城乡和谐稳定，人民富裕安康，率先基本实现社会主义现代化。

实现“十一五”时期发展目标，要全力实施“四大战略”、做到“五个更加注重”。“四大战略”是：产业集聚优化创新战略；生态组团城市发展战略；科教兴市人才强市战略；城乡一元和谐发展战略。“五个更加注重”即：一是更加注重以科学发展观统领发展全局。二是更加注重走新型工业化道路。三是更加注重统筹协调发展。四是更加注重深化改革扩大开放。五是更加注重构建和谐社会。

2006年是“十一五”规划的开局年，是继续推进建设产业强市、文化名城、现代化大城市与富裕和谐佛山取得重要成效的关键一年。做好新一年政府工作意义重大。

2006年工作的指导思想是：以邓小平理论和“三个代表”重要思想为指导，贯彻落实科学发展观，全面贯彻党的十六届五中全会和中央经济工作会议及省委九届八次全会精神，落实市委九届八次全会工作部署，坚持发展第一要务，推动经济增长方式转变和产业结构优化升级，着力自主创新，走新型工业化道路；大力实施科教兴市和人才强市战略，建设文化名城；加快组团城市建设，努力提升城市形象；加强民主法治建设，促进依法行政，建设文明法治社会；切实解决人民群众困难问题，努力推进和谐佛山的建设。

2006年主要预期目标是：生产总值增长12%；单位生产总值能源消耗比上年降低4.5%；环境综合指标达88分；全社会固定资产投资增长13%；社会消费品零售总额增长13%；地方财政一般预算收入增长10%；出口总值增长15%；实际利用外商直接投资增长15%；城市居民人均可支配收入增长6%；农村居民人均纯收入增长5%；居民消费价格总指数升幅控制在3%左右；城镇登记失业率控制在3.5%以内；人口自然增长率控制在5.9‰以下。

为实现上述目标任务，要着重抓好以下十一项工作：

一、坚持走新型工业化道路，加快产业结构优化提升

加大园区建设力度，带动优势产业集约发展。加快推进集约园区发展评估体系的实施，完善基础设施和配套服务体系。加快推进工业园区建设，增强产业载体的集聚力和辐射力。实施大企业带动战略，积极引进和发展产业关联度高、带动力强的项目，引导大企业由制造业向研发和服务业两头延伸，发展以世界500强和跨国企业为龙头的汽配产业群，做强做大家电产业集群，扶持龙头企业进一步发展壮大，带动中小企业向专业化、深加工方向发展。

推动产业结构优化升级，全面提升工业发展水平。大力发展汽车配件、电子信息、显示器件、光机电一体化、新材料、新能源、精细化工、生物医药、环保等高新技术产业和新兴产业，改造提升家用电器、机械制造、金属材料加工及制品、陶瓷建材、纺织服装、食品饮料、塑料制品、家具制造等传统产业。抓好南海西樵纺织创新中心和禅城陶瓷研发检测中心经验的推广，推进一批企业信息化示范工程建设。同时，促进区域产业协调发展。加强全市产业规划和指导，推动全市产业实现有序转移，引导各区发展优势特色产业，提升区域竞争力。

加快以物流业为龙头的现代服务业发展。大力发展与现代制造业相配套的物流业，以及为新型工业化提供服务支撑的现代服务业。继续抓好签约项目的跟踪落实，办好第三届佛山（国际）物流洽谈会。加大对第三方物流企业扶持力度，促进物流业与制造业协调发展。加快商贸、饮食、旅游、会展、信息、金融、中介等为生产和生活服务的现代服务业发展，推动连锁经营、专业配送、代理制等新型业态发展，加快专业批发市场和城市农贸市场的改造升级。积极引进国内外知名品牌企业，培育和发展一批现代化的大型流通企业集团。

切实解决制约经济发展的瓶颈问题。抓好电力、油品、能源等关键要素的供需协调，加快电网电源建设，并实行对峰期地方电厂发电补贴，降低缺电对我市经济发展的影响。加强对成品油市场的有效监管。加强对经济运行的监测，提高调控水平。

二、推动创新型城市建设，增强自主创新能力

加强创新体系建设，增强行业及企业竞争力。抓好“十一五”科技专项和产业技术自主创新规划的实施，引导和鼓励企业建立工程技术研发机构，完善技术创新激励机制，推动企业加大创新投入。依托专业镇，加强行业性、区域性创新体系建设；依托高新科技园区和特色产业基地，建设好科技孵化器；依托省级研究院，加快公共创新平台建设。

加强科技成果转化工作，加快建立多元化产学研合作体系。推动企业与高校、科研院所开展多种形式的合作，扶持高校驻佛山的研究院、院士工作室的工作，促进科技成果产业化。加强各类科技中介服务机构建设，大力扶持生产力促进中心等有特色的中介服务机构建设，逐步形成以市场为导向、产学研为依托，网络化、多功能、专业化的科技中介服务体系。

加强创新支撑服务体系建设，加快建立多元化科技投融资体系。优化自主创新的政务环境，通过政策引导，形成科技与经济紧密结合的有效机制。加大对自主创新的扶持力度，加强以政府投入为引导、市场投入为主体的多元化科技投融资体系建设。加强科技人才队伍建设，健全有利于科技创新和创业人才汇聚的机制。

加强创新能力建设，实施名牌、标准化、专利带动战略。完善创名牌激励机制，扶持各类企业创立名牌，打造“佛山制造”区域品牌，扶持一批有条件的企业向世界名牌冲刺。鼓励和扶持有实力的企业参与行业、国家和国际标准的制定，提高自主创新能力和核心竞争力。以建设全国专利工作试点城市为契机，建立运用知识产权制度促进科技创新的激励机制，加大知识产权的保护教育和宣传力度，支持企业和个人申请国内外发明专利。抓好商标培育工作，努力营造尊重商标、依法注册商标、使用商标、保护商标的商业文化氛围。

三、用“三化”解决“三农”问题，建设社会主义新村镇

推进现代农业发展，加速农业现代化进程。抓好现代农业生态园区和“海峡两岸农业合作试验区”建设，大力发展农业龙头企业，加快农民专业合作经济组织发展，提高农业生产规模化、组织化和产业化程度。实施科技兴农战略，加快发展高质农业、绿色农业、创汇农业、都市农业和观光农业。落实粮食工作责任制，稳定粮食生产。继续推进农产品市场准入制度，健全和完善农副产品质量监管安全体系，加强动植物疫病防控工作。

进一步深化农村改革。今年是落实“农村工作五十条”的首批九个配套政策决定性的一年，要按照“生产发展、生活宽裕、乡风文明、村容整洁、管理民主”的要求，推进社会主义新村镇建设。以基本医疗保险、基本养老保险补贴、最低生活保障为重点，逐步完善与当地经济社会发展水平相适应、城乡对接的农村社会保障体系，重点抓好农村劳动力转移二、三产业以后的社会基本保障和全征地农民基本养老补贴制度的落实。做好农村富余劳动力培训和就业工作，促进有劳动能力、有就业愿望的农村富余劳动力通过培训向二、三产业转移。积极推进农村财务引入中介组织管理改革工作，完善农村股份合作制，增强农村经济发展活力。推进农村信息化建设，发挥信息技术在解决“三农”问题上的积极作用。做好下一步农村改革的调研工作。

加速农村城镇化进程。统筹城乡规划布局，完善和实施镇、村规划，推进城镇化试点工作，走以镇带村、促进村镇协调发展和水平提升的城镇化道路。加快中心城镇建设，推动城市基础设施建设向农村延伸，提高城镇综合承载能力。积极稳妥抓好城中村改造和推进农民公寓建设，引导农村居民转变居住和生活方式，不断提高农村城镇化水平和文明程度。

四、抓好招商引资和民营经济工作，促进内外源型经济协调发展

把握新一轮国际产业转移机遇，提升招商引资的质量和水平。继续把世界500强企业、跨国公司和国内大企业作为招商引资重点，吸引研发中心、营运中心和地区总部进驻佛山。加大对汽配、家电、机械装备、新材料、显示器件等产业和以现代物流业为重点的现代服务业的招商引资力度，争取在吸收外资的质量和水平上有新提升。抓好已签约项目的跟踪和落实，提高履约率、开工率、投产率。

转变外贸增长方式，努力拓展国际市场。积极扩大出口，巩固港澳台、欧美日、东盟等传统市场，继续拓展南美、中东、非洲、印度、俄罗斯等新兴市场。充分利用新兴市场进入门槛低、市场需求量大的特点，推动加工贸易转型升级，拓展市场空间，延长产业链。

认真落实各项扶持政策，做强做大做优民营经济。深入落实《中小企业促进法》和国务院《关于鼓励支持和引导个体私营等非公有制经济发展的若干意见》，完善政策支持体系和服务体系。鼓励和支持民营企业广泛参与区域交流与合作，支持有条件的企业“走出去”，充分利用我市侨务、外事和友好城市的资源，开拓国内外市场。支持和引导行业协会、商会、中介组织等为民营企业发展提供优质服务。鼓励和扶持各类信用担保机构为中小企业融资提供服务，解决中小企业贷款难问题。此外，加强与港澳、珠三角、泛珠三角及国内其他城市的经贸合作与文化交流。

五、加强现代化大城市建设和管理，以新的面貌迎接省运会召开

坚持以规划为指引，加快以中心组团新城区为重点的组团城市建设。突出规划在城市建设中的龙头作用，抓好土地利用总体规划的修编工作。进一步完善规划管理体制，强化管理手段，按照城市总体规划及一系列专项规划的要求，推进规划实施；探索建立组团城市建设管理新机制，加快城市建设。以中心组团建设为引领和示范带动，加快推进其他城市组团建设。

加快以“一环”为重点的交通网络建设。采取有力措施，保质保量完成以“一环”为重点的第一批主干路网工程建设，积极配合做好途经我市的高速公路及铁路项目建设的征地拆迁工作，为各项工程如期开工创造条件。抓好各城市组团基础设施的联通，加快公共交通的整合和发展，完善全市公交服务网络，以良好的城市交通环境，迎接省运会的召开。加快全市港口、码头整合步伐，提升佛山港的竞争力。

加快体育场馆和中心组团新城区基础设施建设。确保岭南明珠体育馆、世纪莲体育中心、游泳馆以及各区体育场馆改造项目在省运会前全部竣工；确保省运会前基本完成中心组团新城区启动区首期道路、综合管沟、水利工程及重点景观工程建设。

加强电力、天然气和水利工程建设。按照电网发展规划，加快一批电源项目建设，完成禅南、藤沙和顺德等输变电工程选址选线和建设工作。推进LNG工程建设。抓好城乡水利防灾减灾工程建设，确保全部建设任务的完成。

按照现代化大城市管理思路，强化城市管理。加强城市管理机构和队伍的建设，建立管理制度，加大执法力度，提高管理水平；以交通道路和中心城区市容市貌管理为主要内容，突出重点，抓好整治，努力营造安全、有序、文明、整洁的城市环境，进一步提升城市形象。

六、进一步深化各项改革，营造体制机制新优势

深化行政管理体制改革。进一步转变政府职能，强化政府社会管理和公共服务职能，加快构建行为规范、运转协调、公正透明、廉洁高效的行政管理体制。进一步推进行政审批制度改革与创新，建设好市、区行政服务中心，推动“一站式”电子政务建设和行政审批电子监察系统建设，完善行政审批管理监督机制。抓好行政机关、事业单位经营性资产清理工作。推进综合行政执法改革试点工作。做好事业单位分类改革的准备，全面推行事业单位人员聘用制度。加强对行业协会和中介机构的培育和监管。

推进公有企业和国有资产监督管理体制改革。继续推进公有企业股份制改造，大力发展资本市场，鼓励和支持企业上市融资，做好上市企业股权分置改革工作，加大劣势企业退出市场力度。进一步完善国有资产监管体系，创新公有企业领导人员管理方式，探索市、区国有资产监管体系衔接，实现国有资产有效监管。

深化投融资体制改革。完善投资核准和备案制度，规范政府投资行为，强化政府投资决策责任。抓好已签约项目的履约和落实，认真做好新项目的策划、包装和推介工作。抓紧实施城市可经营项目监督管理办法，明确监督管理责任，确保公共产品安全和有效供给。

七、促进资源环境相协调，建设资源节约型和环境友好型社会

抓好创建国家环保模范城各项任务的落实，建设环境友好型社会。以抓好城乡河涌污染综合整治为突破口，以全力改善环境空气质量和重点区域的城市环境面貌为主要内容，加快推进佛山水道和内河涌的综合整治，提高生活污水处理率和工业污水排放达标率，改善水环境质量，确保饮用水安全。加大对工业二氧化硫、烟尘粉尘和机动车尾气污染治理力度，改善重点区域环境空气质量。加快第二饮用水源供水工程的建设步伐。加强环境监督执法，进一步完善污染源在线监控，提高污染控制与处置能力以及环境管理水平。加快“绿色佛山”和生态型城市、生态示范区、绿色社区、全国环境优美乡镇建设，推进洁净、碧水、蓝天、绿化、美化、安静工程建设，努力提升全社会环境意识，力争通过创建国家环保模范城市的验收。做好地质灾害防治工作，促进人与自然环境的和谐共处。

积极发展循环经济，建设资源节约型社会。做好节约型社会试点城市工作。强化对城镇土地、能源、水等资源的管理，推动资源节约和合理高效利用。制定我市有关行业、企业资源节约的目标，完善考核体系。加快新能源和环保产业发展，大力推广清洁生产和清洁能源应用，加大节能产品开发和技术改造投资力度，支持一批资源节约型技术开发和改造项目，提高资源节约整体水平。

八、推进文化名城建设，提升城市品位和综合竞争力

推进教育现代化建设，提高教育发展水平。高质量高水平普及九年义务教育和普通高中教育，优化学前教育，促进民办教育和社区教育，大力发展职业教育，加快发展高等教育，推进以发展高等职业教育为重点的佛山大学园区建设。重视教育公平，推进区域教育均衡发展。重视并抓好外来务工人员子女的教育。积极创造条件，探索开展免费义务教育试点工作。实施人才强教工程，建设高素质师资队伍。抓好教育现代化建设，不断提升教育信息化和教育管理水平。

推进人才强市战略，构筑人才资源高地。完善有利于优秀人才脱颖而出和充分发挥人才作用的体制机制，落实各项政策措施，构建有利于各类人才实现可持续发展的公共服务平台。加强党政人才、企业经营管理人才和专业技术人才队伍建设，实施“优秀人才培养工程”，进一步建设好企业博士后工作站。实行产业引进与人才引进相结合战略，积极引进国内外中高端人才，为创建创新型城市提供坚实的人才基础。大力发展并规范管理人才市场，加强区域间人才合作，促进人才智力的广泛交流与合理利用。

深化文化体制改革，促进文化与经济进一步融合。总结推广承办亚洲文化部长论坛暨第七届亚洲艺术节的经验和做法，大力发展具有佛山特色的文化事业和产业，打造知名的文化品牌。坚持继承和创新相结合，加强对佛山历史人文资源的保护和开发利用，进一步加强宣传推介力度，弘扬陶艺、粤剧、武术、民间艺术、饮食等传统文化，打造具有佛山特色的知名旅游品牌，增强文化和旅游竞争力。深化文化体制改革，推动文化与经济的进一步融合。积极构建城市公共文化服务体系，加强公共图书馆、公益性博物馆等文化基础设施建设，保障市民享受文化和参与文化的权利。提高文化产业的市场化程度，培育壮大新闻出版、广播电视、音像、演艺、文化、旅游等有佛山特色的文化产业。

全力承办好第十二届省运会。动员全市力量，认真做好省运会筹备工作，努力实现“精彩省运、活力佛山”的目标。同时，办好第五届省残运会。狠抓备战，促进竞技体育运动水平的提升。以承办省运会为契机，进一步推动群众性体育运动的蓬勃开展。加强公共体育设施和群众性健身场所建设，为全民健身提供有利条件。

加快卫生、人口与计生事业发展。推进基层医疗卫生网络建设，大力发展社区卫生服务。加强公共卫生体系建设，健全三级卫生监督和疾病控制网络。完善疾病预防控制、医疗救治和卫生执法监督体系，强化对食品、药品、餐饮卫生的监管。高度重视高致病性禽流感等传染病的防治。大力发展中医事业和中药产业，打造国内外知名的中医药品牌。深化药品和医疗服务价格改革，降低群众医疗费用负担。进一步抓好人口与计划生育工作。开展爱国卫生运动，增强全民卫生意识，提高城乡卫生整体水平。

九、加强财政金融工作，保障和促进经济健康发展

严格依法治税，加强收入征管。依法强化税收征管，抓好公平税负，改革和完善征管体制。健全财税收入完成情况分析联席会议制度，加强沟通，减少漏征漏管。努力拓宽收入来源，积极开拓非税收入。进一步建立协税护税网络，全面加强税收征管。

健全公共财政体制，优化财政支出结构。进一步调整和优化财政支出，集中财力保证教育、科技、环保、社会保障、公共卫生、就业和再就业以及“十项民心工程”等重点支出的需求，保证交通、能源、水利、城市基础设施、省运会场馆等工程建设的资金投入。大力压缩一般性支出，严格控制各项庆典活动费用开支。规范政府采购预算管理，强化政府采购监督。加强政府债务管理，减少财政风险。

规范财政管理，强化财政监督。继续细化部门预算，全面铺开国库集中支付改革，扩大政府采购的范围和规模，加强“收支两条线”管理，推进绩效评价工作，确保财政资金的安全、高效、规范运转。加强财政管理，逐步建立健全市对区、区对镇（街）的财政监督约束机制。完善财政查询监督系统，强化人大、审计对财政资金运行的在线监督。

加强金融监管，维护金融秩序。制定金融业发展规划，以规划引领金融业的良性发展。建立金融联席会议制度，加强业内交流沟通。推进“信用佛山”建设，加强合同监管，提高企业信用，密切银企关系。全面推进金融生态示范市建设，加快票据交易中心建设，大力发展信用担保公司，发挥银团贷款的作用。继续推进农村信用社改革。

十、推进民主法制建设和社会事业发展，构建富裕和谐佛山

加强精神文明建设。深入实施《公民道德建设实施纲要》，进一步开展“爱国、守法、诚信、知礼”现代公民教育活动，加强社会公德、职业道德和家庭美德教育，加强和改进未成年人思想道德建设，倡导科学文明健康的生活方式，鼓励社会各界参与志愿者活动，树立现代社会文明风尚。加强全民国防教育，深入开展双拥共建活动。以创建文明城市为抓手，以创建文明行业、社区、街道、乡村为重点，积极开展全方位的文明创建活动，推动全社会文明诚信建设，建设学习型社会。

推进民主法制建设。坚持向人大及其常委会报告工作、向政协通报情况的制度，自觉接受人大及其常委会和政协的监督。认真办理人大议案、人民代表建议和政协提案。充分听取民主党派、工商联、无党派人士和人民团体参政议政意见。落实与民主党派的对口联系制度。发挥工会、共青团、妇联等人民团体的桥梁纽带作用。以政务公开信息化、规范化、制度化，推进政府决策的科学化、民主化和程序化，进一步健全决策咨询、重大决策及建设项目社会参与和社会听证制度，拓展市民参与政务活动的渠道。切实保障社会公众的知情权、参与权和监督权，充分发挥新闻媒体的舆论监督作用。重视并加强社区及基层组织的民主法制建设。

努力营造和谐安宁环境，维护社会稳定。按照国务院和省的要求，加快突发公共事件应急机制建设，有效提升处置突发事件的能力。巩固和推广科技强警示范市建设成果，深入开展平安和谐社区建设，加强对流动人员和出租屋的管理，依法严厉打击各类违法犯罪活动，扫除“黄赌毒”等社会丑恶现象和各类黑恶势力，切实保障人民群众生命财产安全。加强国家安全工作。深入整顿和规范市场秩序，营造良好的市场环境。强化安全生产管理责任制，进一步加大交通、生产、消防、森林防火和公共卫生等安全事故防范和处置力度，防范和化解重特大安全事故。加强对外来务工人员的权益保护，强化工资支付监控制度，规范企业工资支付行为，妥善解决劳资纠纷。

健全城乡就业服务体系。进一步完善就业扶持政策，改善就业环境，健全市、区、镇（街）三级公共就业服务机构和职业培训体系，搭建城乡统一的就业服务平台，推进就业和再就业工程建设。积极引导城镇下岗失业人员改变择业观念，千方百计提供更多就业岗位。建立城乡一体化的市场就业机制，促进城乡劳动力合理流动。

进一步完善社会保障体系。依法扩大社会保险覆盖面，做实做好养老保险个人账户，完善社会保险基金监管制度。提高社会保险统筹水平，增强社会保险基金调剂能力。加快进城务工人员纳入城镇职工社会保险体系步伐，确保劳动者平等享有社会保障权利。完善失业保险制度，增强失业保险促进就业功能。完善多层次、广覆盖的基本医疗保障体系。加快建立城乡一体的社会救助体系，为困难群众提供更好的生活保障。做好企业退休人员社会化管理服务工作。

不断改善人民群众生活，维护社会公平。扎实推进“十项民心工程”，帮助困难群众解决实际问题。加大帮扶力度，加强残疾人就业、基本生活和福利事业，进一步完善扶贫助学政策体系，切实解决城乡低收入家庭的住房、医疗和子女上学等困难问题。加强妇女儿童工作。高度重视社会不安定因素的预防处置工作，采取有效措施解决征地拆迁、拖欠工资等方面存在的突出问题。加强信访和人民调解工作，畅通诉求渠道，及时处理群众反映的突出问题，有效化解人民内部矛盾。强化法律援助工作，引导群众运用法律手段解决矛盾纠纷。

十一、加强和改进政府工作，提高依法行政水平

坚持依法行政，提高理政水平。进一步贯彻落实《行政许可法》和《全面推进依法行政实施纲要》，加强依法行政工作。健全行政执法责任制，提高城市综合执法水平。加强学习，提高政府部门和各级领导干部科学理政、民主施政、依法行政的能力和水平。

加强政风建设，强化责任意识。加强行政效能投诉中心的建设，强化对政府部门的行政效能监察，完善并落实行政效能岗位责任制。抓好《公务员法》的实施，加大公务员教育培训力度，完善公务员考核激励机制，不断提高公务员队伍能力和素质。

加强勤政廉政建设，提高拒腐防变能力。建立健全惩治和预防腐败体系，进一步探索从源头上预防和解决腐败问题的有效途径。抓好政府系统党风廉政建设，提高领导干部拒腐防变能力。强化对工程建设领域的监督检查，确保“资金安全，工程优质，干部优秀”。开展民主评议行风活动，纠正损害群众利益的不正之风，做到理政为民。坚决查办各类违纪违法案件，严厉惩处腐败分子。规范和完善政务、厂务、村务公开工作，拓宽群众有序的政治参与和监督渠道。

各位代表！我们正处在全面建设产业强市、文化名城、现代化大城市与富裕和谐佛山的重要时期，使命光荣，任重道远。让我们高举邓小平理论和“三个代表”重要思想伟大旗帜，牢固树立和认真落实科学发展观，在省委、省政府和市委的正确领导下，同心同德，抓实机遇，与时俱进，锐意创新，为“十一五”发展起好步、开好局，为率先基本实现社会主义现代化作出新的更大贡献！
